# Data on localization of coxsackievirus and adenovirus receptor (CAR) in prenatal and adult rat olfactory, intestine, pancreas, liver, ovary, and testis

**DOI:** 10.1016/j.dib.2019.103797

**Published:** 2019-03-01

**Authors:** Mo Chen, Saishu Yoshida, Takako Kato, Yukio Kato

**Affiliations:** a Division of Life Science, Graduate School of Agriculture, Meiji University, Kawasaki, Kanagawa, 214-8571, Japan; b Institute for Endocrinology, Meiji University, Kawasaki, Kanagawa, 214-8571, Japan; c Department of Life Science, School of Agriculture, Meiji University, Kawasaki, Kanagawa, 214-8571, Japan

## Abstract

The data in the present article are related to the previous article entitled “Coxsackievirus and adenovirus receptor-positive cells compose the putative stem/progenitor cell niches in the marginal cell layer and parenchyma of the rat anterior pituitary” (M. Chen et al. 2013). The data describe the characteristic localization in the immature cells of the prenatal and adult tissues beyond the germ layer. Germ cells and the reproductive tissues of both sexes showed distinct intracellular polarities of CAR: apical, basolateral, and pericellular in the immature cells of the embryo and adult tissues. In addition, the data describe on localization of CAR in the methimazole-induced damage of olfactory epithelium tissue. The data show that the CAR-_immuno-positive cells at the apical side of the olfactory epithelium disappeared following methimazole treatment and reappeared in the regenerating stem/progenitor cells (positive for KI67 and E-cadherin) of the basal layer with basolateral expression.

**Table:** Specifications table

Subject area	*Biology*
More specific subject area	*Developmental biology*
Type of data	*Immunohistochemistry*
How data was acquired	*Immunofluorescence images were obtained by fluorescence microscopy (Keyence BZ-9000).*
Data format	*Tables, figures*
Experimental factors	*Immunohistochemistry was performed for CAR, the cell division marker KI67,and the stem/progenitor cell markers SOX2 and E-cadherin.*
Experimental features	*Tissues were prepared from prenatal and adult rats and methimazole-induced olfactory-damaged rats. Sections were fixed with 4% (w/v) paraformaldehyde. After immuno-reaction with primary antibodies, fluorescein isothiocyanate-, Cy3-, or Cy5-conjugated secondary antibodies were used for detection.*
Data source location	*Kawasaki, Kanagawa, Japan*
Data accessibility	*Data are within the present article*
Related research article	*M. Chen, T. Kato, M. Higuchi, S. Yoshida, H. Yako, K. Kanno, Y. Kato. Coxsackievirus and adenovirus receptor-positive cells compose the putative stem/progenitor cell niches in the marginal cell layer and parenchyma of the rat anterior pituitary. Cell and Tissue Research. Dec; 2013;354:823-36*[1].

**Table:** 

**Value of the data** • The data would provide a platform to further explore and understand the role of a common receptor for coxsackievirus and adenovirus (CAR) in tissue development. • The data on the localization of CAR in the cells of the prenatal and adult rat tissues beyond the germ layer can be useful for researchers interested in tissue differentiation and development. • The data on the apical and basolateral localization of CAR in differentiating tissues can be useful for investigators interested in molecular mechanism of stem/progenitor cell function. • The present data that CAR- and E-cadherin-expressing stem/progenitor cells are involved in the regeneration of the olfactory epithelium following methimazole-induced damage could be valuable in understanding olfactory epithelial regeneration.

## 1 Data

**Fig. 1:**
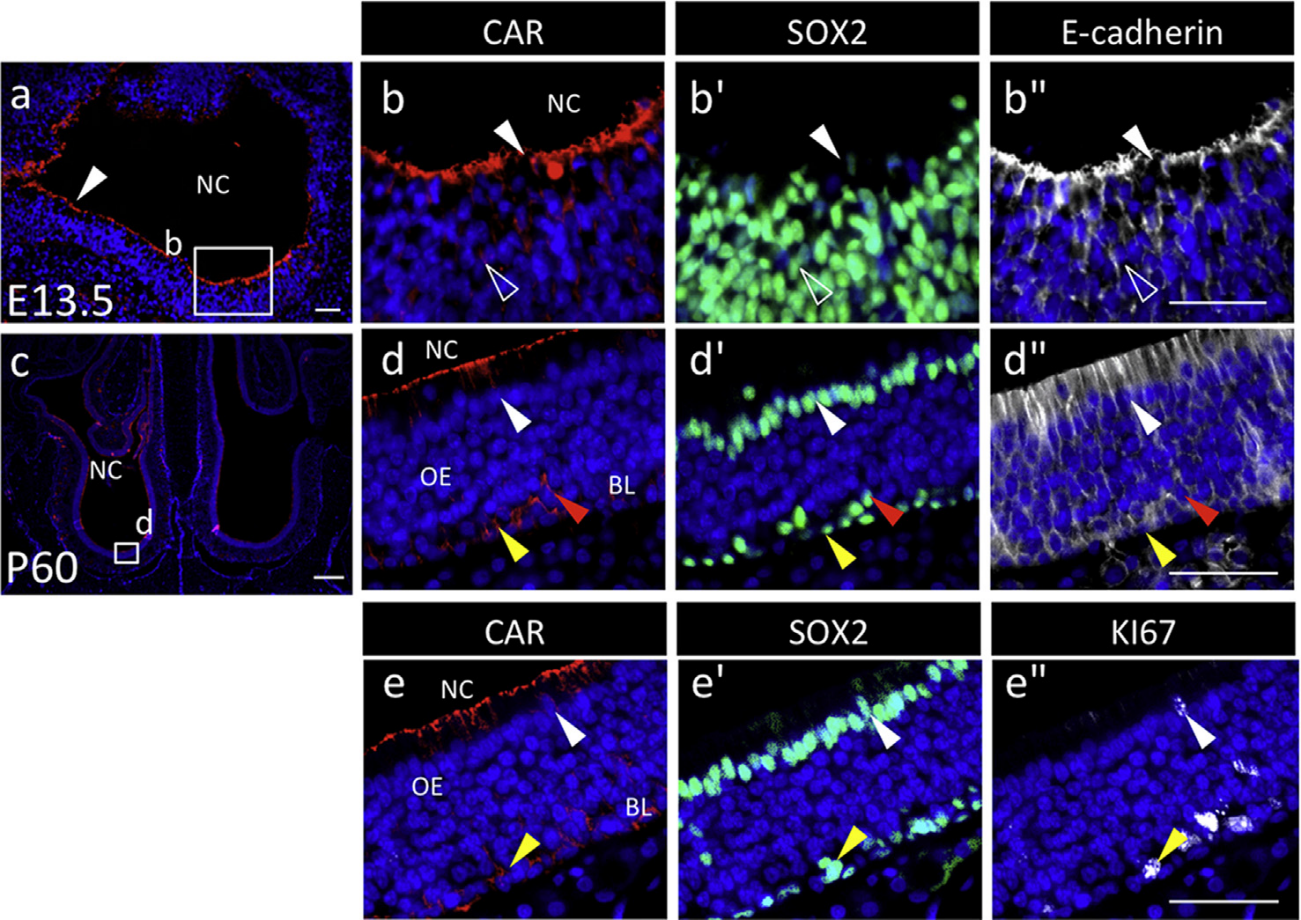
Immunohistochemistry of coxsackievirus and adenovirus receptor (CAR) in the olfactory tissues originating from the surface ectoderm. Immunostaining with CAR (Cy3, *red*), SOX2 (FITC, *green*), E-cadherin (Cy5, false-color in *white*), and KI67 (Cy5, false-color in *white*) and staining of nuclei with DAPI (*blue*) was carried out for a sagittal plane of prenatal olfactory tissues on embryonic day 13.5 (E13.5) (**a**) and a coronal plane of adult olfactory tissues on postnatal day 60 (P60) (**c**). Boxed regions in **a** and **c** are enlarged in **b**–**b''** and **d**–**d''**, respectively, and showed merged images with DAPI. Images in **e**–**e''** were made with an adjacent section of **d**–**d''**. CAR-positive cells are indicated with *white arrowheads* (in the surface cell layer), *open arrowheads* (in the parenchyma), *yellow arrowheads* (in the basal cells), and *red arrowheads* (in the secondary layer cells of basal lamina). *NC* nasal cavity; *OE* olfactory epithelium; *BL* basal lamina. *Scale bars* 50 μm (**a, b'', d, e'**'), or 500 μm (**c**).

Data on immunohistochemistry for coxsackievirus and adenovirus receptor (CAR), which plays multifold functions [1], [2], [3], [4], [5], show its localization in several rat tissues beyond the germ layer as summarized in Table 1. Images of the prenatal olfactory area originating from the surface epithelium of the ectoderm show coexistence with stem/progenitor markers, E-cadherin and SOX2, and with a cell division marker, KI67, in some of the CAR/SOX2-double positive cells (Fig. 1).

**Table 1:** Summary of CAR-positive cell.

		Prenatal			Postnatal		
		CAR-positive cells	Localization of CAR	Colocalization	CAR-positive cells	Localization of CAR	Colocalization
Ectoderm	Olfactory	surface cells	apical	E-cad, SOX2	surface cells	apical	E-cad, SOX2
		parenchymal cells	basolateral	E-cad, SOX2	basal cells	basolateral	E-cad, SOX2
					secondary layer cells (partial)	pericellular	E-cad, SOX2
Endoderm	Intestine	innermost cell layer cells	apical	E-cad	simple columnar epithelia	apical	E-cad
		a part of inner layer cells	basolateral	E-cad	intestinal crypts	apical	E-cad
					intestinal villus	basolateral	E-cad
	Pancreas	primitive epithelium cells	apical	E-cad	pancreatic ductal cells	apical	E-cad
		cells facing the lumens	apical	E-cad	intercalated ductal cells	apical	E-cad
	Liver	parenchymal cells	apical	E-cad	mono-layered bile ductal cells	apical	E-cad
Mesoderm	Overry	NA			immature, primary, secondary, tertiary vesicular follicles oocytes	pericellular	
					Graffian follicle	pericellular	E-cad
					granulosa cells	pericellular	E-cad
					simple squamous epithelial cells simple cuboidal epithelial cells stratified cuboidal epithelial cells	apical apical/batholateral apical	E-cad
	Testis	NA			spermatogonia, spermatocytes and elongated spermatids	convex surface at the particular stages	NA
		NA			mesenchymal cells Leydig cells	apolar	NA
					spermatozoa	acrosome	NA

CAR: coxsackievirus and adenovirus receptor, E-cad: E-cadherin, SOX2: sex determining region Y-box 2, NA: not available.

**Fig. 2:**
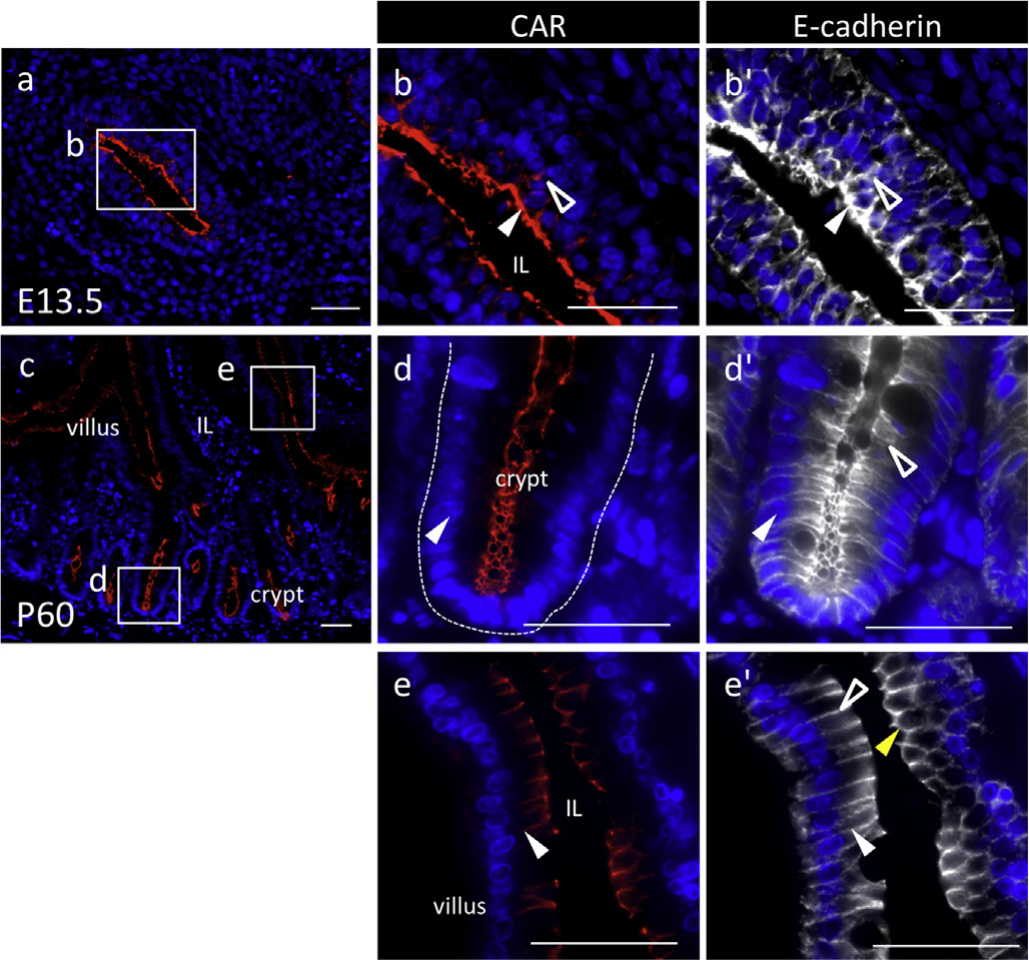
Immunohistochemistry of coxsackievirus and adenovirus receptor (CAR) in the intestine originating from the endoderm. Color codes are the same as those in Fig. 1. Merged images of CAR or E-cadherin with DAPI in a sagittal plane from rat prenatal intestine on E13.5 (**a**) and adult intestine at P60 (**c**) are shown. Boxed regions in **a** and **c** are enlarged in **b**–**b''**, and in **d**–**d''** and **e**–**e''**, respectively, and showed merged images with DAPI. CAR/E-cadherin-double positive cells are indicated with *white arrowheads*. Basolateral and pericellular localizations of CAR are indicated with *open arrowheads* and *yellow arrowheads*, respectively. *IL* intestinal lumen. *Scale bars* 50 μm.

**Fig. 3:**
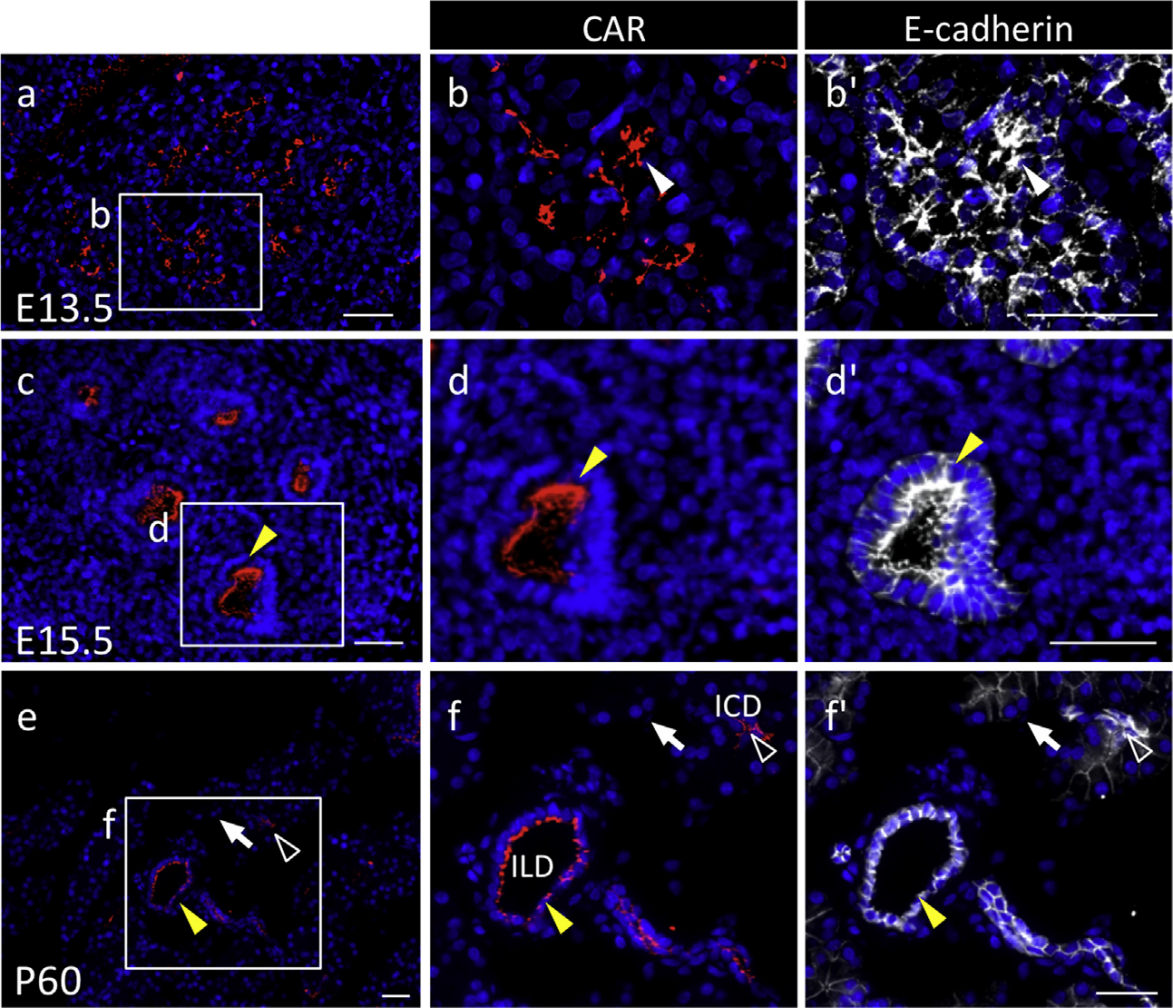
Immunohistochemistry of coxsackievirus and adenovirus receptor (CAR) in the pancreas originating from the endoderm. Color codes are the same as those in Fig. 1. Merged images with CAR or E-cadherin and DAPI in a sagittal plane for E13.5 (**a**), E15.5 (**c**), and P60 (**e**) are shown. Boxed regions in **a**, **c**, and **e** are in **b**–**b’**, **d**–**d’**, and **f**–**f’**, respectively, enlarged and showed merged images with DAPI. CAR-positive cells are indicated with *white arrowheads* (in the duct), *open arrowheads* (in the interlobular duct), and *yellow arrowheads* (in the intercalated duct). *Arrows* indicate the exocrine cells. *ILD* interlobular duct; *ICD* intercalated duct. *Scale bars* 50 μm.

**Fig. 4:**
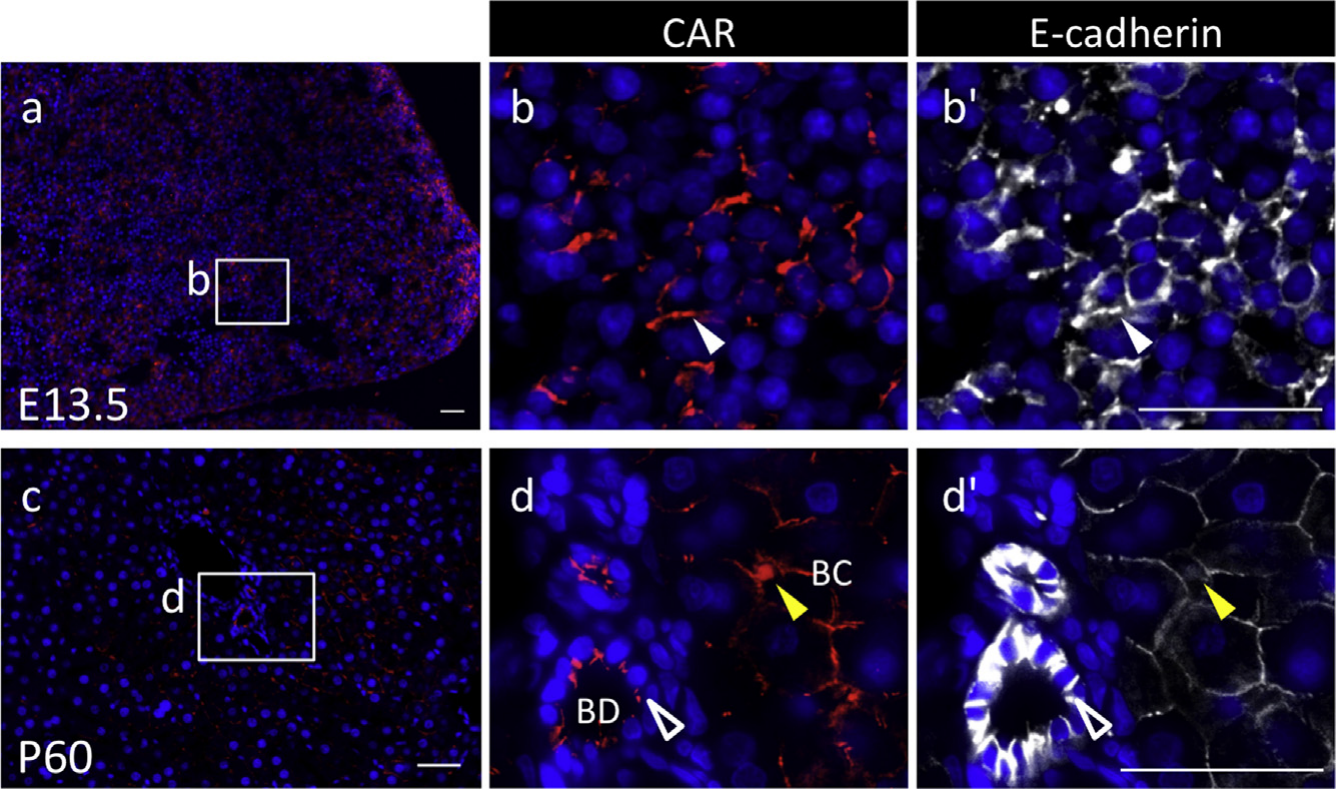
Immunohistochemistry of coxsackievirus and adenovirus receptor (CAR) in the liver originating from the endoderm. Color codes are the same as those in Fig. 1. Merged images with CAR or E-cadherin and DAPI in a sagittal plane for E13.5 (**a**–**b'**) and P60 (**c**–**d'**) are shown in **a**. Boxed regions in **a** and **c** are enlarged in **b**–**b’** and **d**–**d’**, respectively. CAR-positive cells are indicated with *white arrowheads* (in the parenchyma), *open arrowheads* (in the bile duct), and *yellow arrowheads* (in the bile canaliculi). *BC* bile canaliculi; *BD* bile duct. *Scale bars* 50 μm.

Data on the endodermally-derived tissues, such as the intestine, pancreas, and liver, show CAR-positive signals with apical, basolateral, and pericellular polarities and coexistence with E-cadherin (Fig. 2, Fig. 3, Fig. 4).

**Fig. 5:**
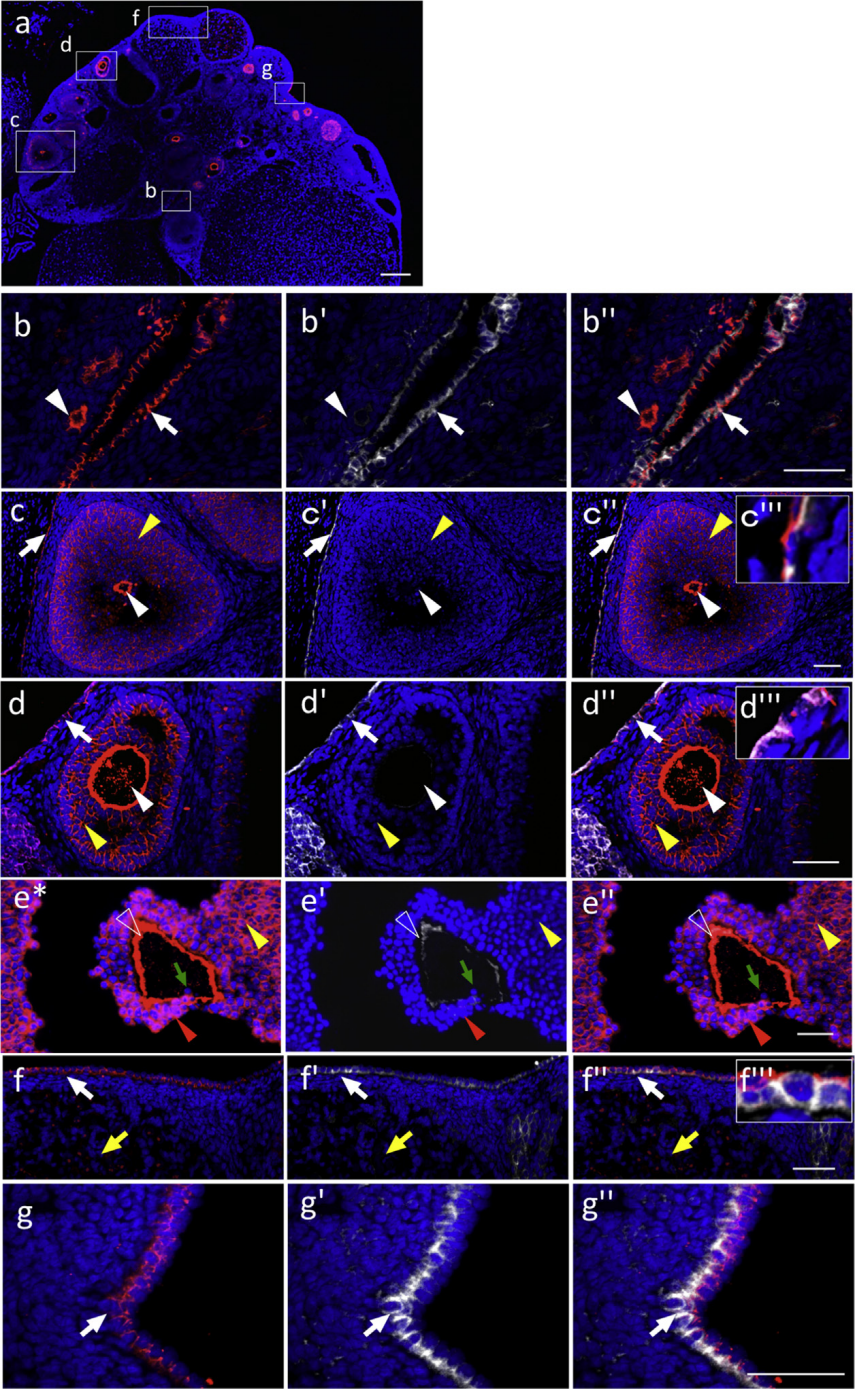
Immunohistochemistry of coxsackievirus and adenovirus receptor (CAR) in ovary tissues originating from the mesoderm. Color codes are the same as those in Fig. 1. Merged image of CAR with DAPI in a sagittal plane for P60 is shown in **a**. Boxed regions in **a** are enlarged in **b** (developing, immature, and primary follicles), **c** (secondary follicles), and **d** (tertiary vesicular follicles), **f** (atretic follicle), and **g** (surface epithelial cells). Enlarged image of the Graafian follicle is shown in **e** from a different section. CAR-positive signals are indicated with *white arrowheads* (follicle), *open arrowheads* (pellucid zone), *yellow arrowheads* (granulosa cells), *red arrowheads* (cumulus cells), and *yellow arrows* (atretic follicle). Oocytes, negative for CAR, are indicated with *green arrows. White arrows* indicate germinal epithelial cells (CAR/E-cad-double positive) together with an enlarged image inset (**c’’’**, **d’’’, f’’’**): simple squamous epithelial cells (**b**, **c**), and simple (**d**) and stratified (**f**) cuboidal epithelial cells. *Scale bars* 500 μm (**a**), or 50 μm (**b''–g''**).

**Fig. 6:**
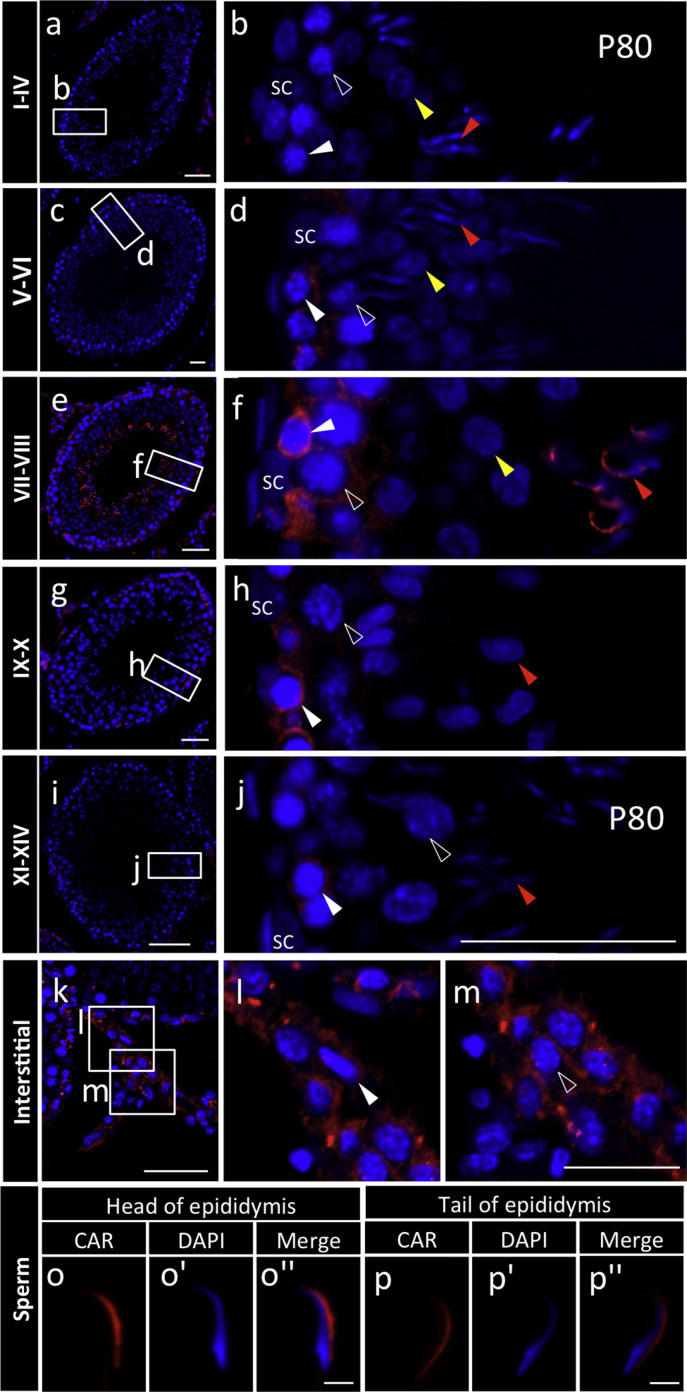
Immunohistochemistry of coxsackievirus and adenovirus receptor (CAR) in the testis and sperm. Coronal sections of the testis (fixed in Bouin fixative) and sperm prepared from epididymis (fixed in paraformaldehyde) on P60 were performed for immunostaining with CAR (Cy3, *red*) and nuclear staining with DAPI (*blue*). Merged images for every stage of seminiferous tubule are shown in the left panels and enlarged images are shown in the right panels. *White, open, yellow,* and *red arrowheads* in **b**–**j** indicate spermatogonia, spermatocytes, round spermatids, and elongated spermatids, respectively. *White* and *open arrowheads* in **l**–**m** indicate mesenchymal cells and Leydig cells, respectively. Image of sperm separated from head (**o**–**o''**) and tail of epididymis (**p**–**p''**) are shown. *SC* Sertoli cell. Scale bars 50 μm (**a**, **c**, **e**, **g**, **i**, **k**, **m**).

Data on the mesodermally-derived adult tissues show that CAR was positive in the developing immature, primary, secondary, and tertiary vesicular follicles, except for atretic follicle (Fig. 5f), and in the pericellular of oocytes and pellucid zone of the Graafian follicle (Fig. 5e). CAR-signals are observed with polarized locations along with E-cadherin in the three ovarian surface epithelial cell types (Fig. 5c’’’, d’’’, f’’’) covering the ovary containing undifferentiating cells [6], [7]. Data on the adult testis showed stage-dependent localization of CAR during spermatogenesis (Fig. 6a–j) and in the testicular interstitium, some of the mesenchymal cells and Leydig cells (Fig. 6k–m). Acrosome of spermatozoa in the epididymis was positive for CAR (Fig. 6o–p”).

**Fig. 7:**
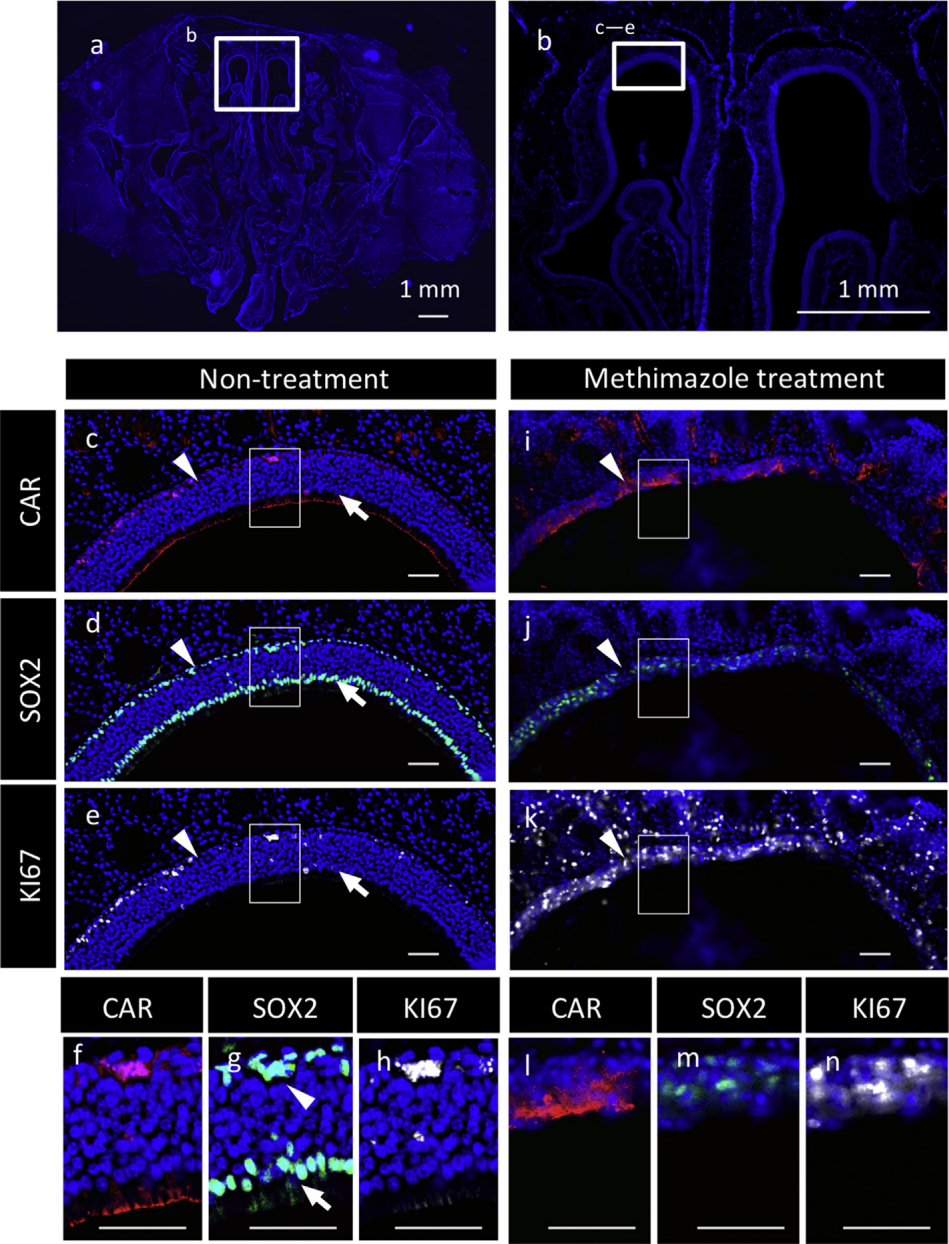
Immunohistochemistry for CAR, SOX2, and KI67 in the olfactory epithelium. Nuclear staining with DAPI (blue) and immunohistochemistry for CAR (Cy3, red), SOX2 (FITC, green), and KI67 (Cy5, white) were performed for non-treatment (**a**–**h**) and methimazole-treatment (**i**–**n**) rats. Boxed areas in **c**–**e** and **i**–**k** are enlarged in **f**–**h** and **l**–**n**, respectively. Arrowheads and arrows indicate basal cells and surface cells, respectively. Scale bars, 1 mm (**a**, **b**), 50 μm (**c**–**e**, **f**–**g**).

**Fig. 8:**
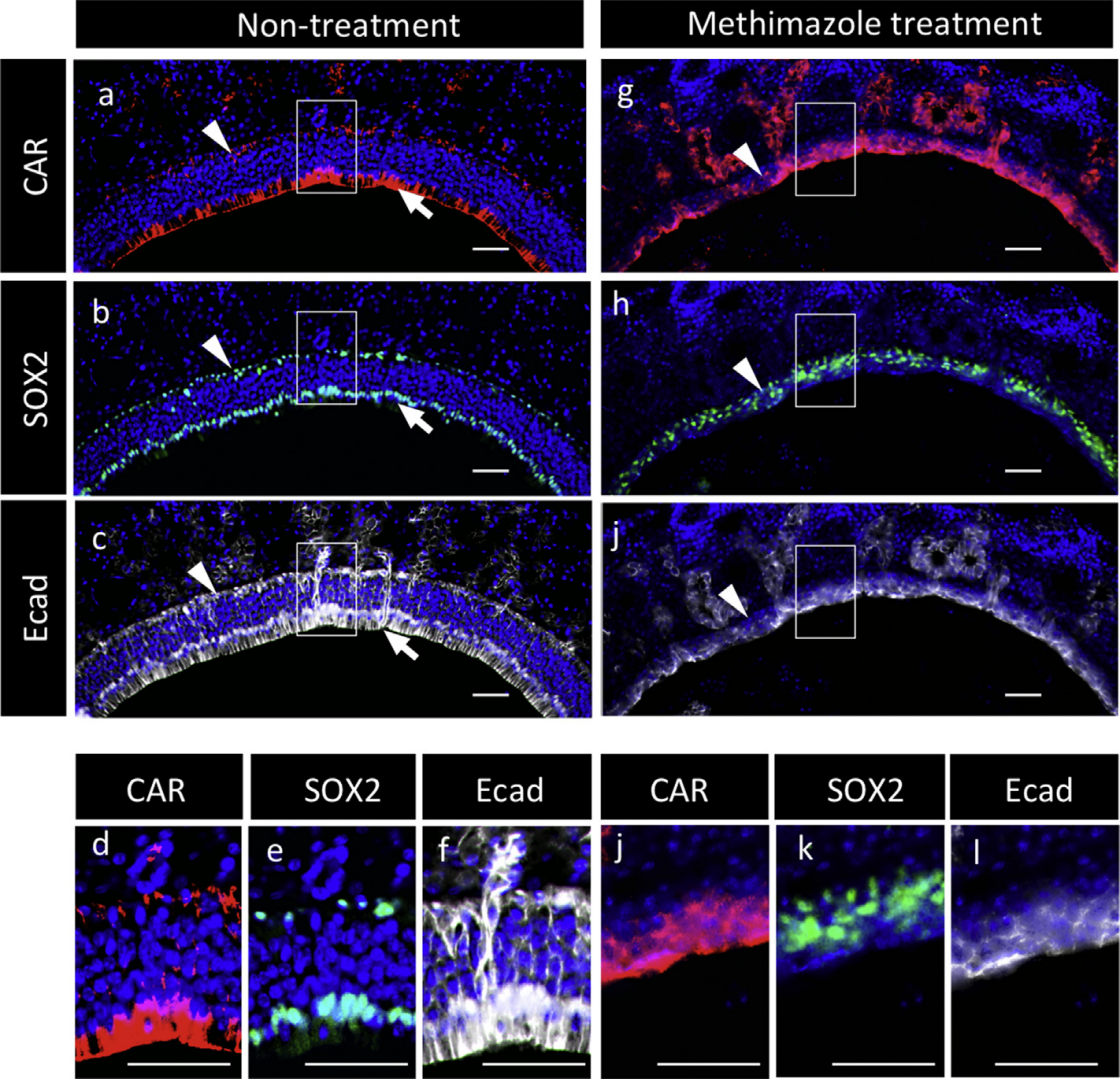
Immunohistochemistry for CAR, SOX2, and E-cadherin in the olfactory epithelium. Color images and indications are the same as in Fig. 7, except for **e**, **h**, **k**, and **n** (Cy5, white, image for E-cadherin). Boxed areas in **a**–**c** and **g**–**i** are enlarged in **d**–**f** and **j**–**l**, respectively. Scale bars, 50 μm.

Data on CAR after methimazole treatment to damage the olfactory mucosa [8] and to regenerate with neural crest-derived stem cells [9] show the disappearance of the apically CAR-positive cell layer and the appearance of a basolaterally CAR-positive layer of the stem/progenitor cells positive for SOX2, KI67, and E-cadherin (Fig. 7, Fig. 8).

## 2 Experimental design, materials and methods

### 2.1 Animals

Male Wistar-Imamichi strain rats were used. Breeding of rats and sampling of brains are described in the previous papers [1]. The present experimental design was approved by the Institutional Animal Care and Use Committee, Meiji University and was performed in accordance with the NIH Guidelines for the Care and Use of Laboratory Animals.

Lesions in the olfactory epithelium were induced according to a previously published method [10]. Methimazole (63760 Fluka, Sigma–Aldrich, Saint Louis, MO, USA) was diluted at 5 mg/ml in 0.9% NaCl and injected intraperitoneally into 12-week-old rats at a concentration of 50 mg/kg of body weight. The rats were euthanized at 1 day after the methimazole injection. Rats were sacrificed by exsanguination from the right atrium under deep pentobarbital anesthesia (40 mg/kg) and then perfused with 4% paraformaldehyde in 0.02 M HEPES buffer (pH 7.4) for experiments.

### 2.2 Immunohistochemistry

Procedures of an antigen retrieval, fixation, and immunostaining were performed as described previously [1] using primary antibodies and secondary antibodies under the conditions listed in Table 2.

**Table 2:** List of antibodies.

A. List of primary antibodies				
Primary antibody	Species	Isotype	Working dilution	Vendor (Area)
CAR	Rabbit	IgG	1:500	Santa Cruz Biotechnology (Dallas, Tex., USA)
SOX2	Goat	IgG	1:400	Neuromics (Edina, Minn., USA)
E-cadherin	Mouse	IgG	1:200	BD Biosciences (San Jose, Calif., USA)
KI67	Mouse	IgG	1:200	BD Biosciences

B. List of secondary antibodies				
Secondary antibody	Species/Isotype	Label	Working dilution	Vendor (Area)
Anti-rabbit IgG	Donkey/IgG	Cy3	1:500	Jackson ImmunoResearch (West Grove, Pa., USA)
Anti-goat IgG	Donkey/IgG	FITC	1:500	Jackson ImmunoResearch
Anti-mouse IgG	Donkey/IgG	Cy5	1:400	Jackson ImmunoResearch
Anti-mouse IgG	Donkey/IgG	FITC	1:500	Jackson ImmunoResearch
